# Supplementary material for: Global tree growth resilience to cold extremes following the Tambora volcanic eruption
Source: Nat Commun. 2023 Oct 19;14:6616. doi: 10.1038/s41467-023-42409-w (PMC10587176; doi:10.1038/s41467-023-42409-w)
Supplement: Supplementary file 1 — Supplementary Information [file 41467_2023_42409_MOESM1_ESM.pdf]

Supplementary Information for

Global tree growth resilience to cold extremes following the Tambora volcanic eruption

Shan Gao, J. Julio Camarero, Flurin Babst, Eryuan Liang*

* **Correspondence:** Eryuan Liang. Email: liangey@itpcas.ac.cn

This PDF file includes:

Supplementary Fig. 1 to 5
Supplementary Table 1

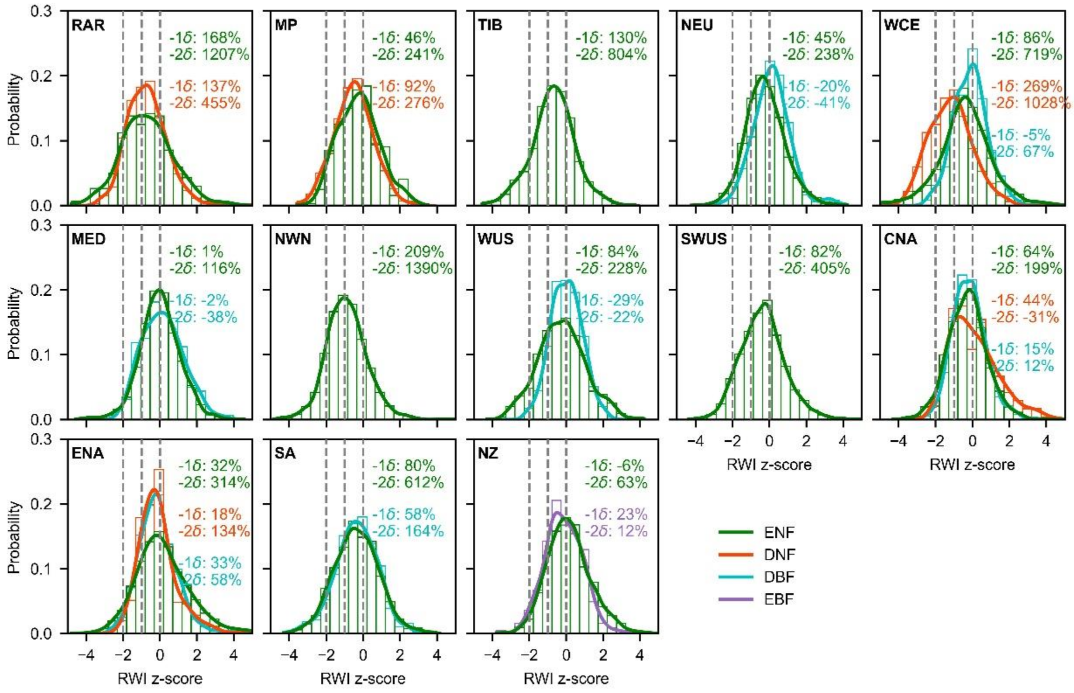

Supplementary Fig. 1 | Change of growth extremes after 1809 and 1815 volcanic eruptions (1809-1824) compared to 1759-1808. The percentages in each panel showed the increased ratio of the percentage of RWI below -1/-2 standard deviation (σ) (deshed lines) in 1809-1824 relative to 1759-1808. ENF, DNF, DBF and EBF represent evergreen needleleaf forests, deciduous needleleaf forests, deciduous broadleaf forests and evergreen broadleaf forests, respectively. RAR, NEU, WCE, MED, MP, TIB, NWN, WUS, SWUS, CNA, ENA, SA and NZ represent Russian-Arctic, northern Europe, western and central Europe, the Mediterranean region, the Mongolian Plateau, the Tibetan Plateau, northwestern North America, the west coast of US, the Southwestern U.S., central North America, eastern North America, the southern Andes and New Zealand, respectively.

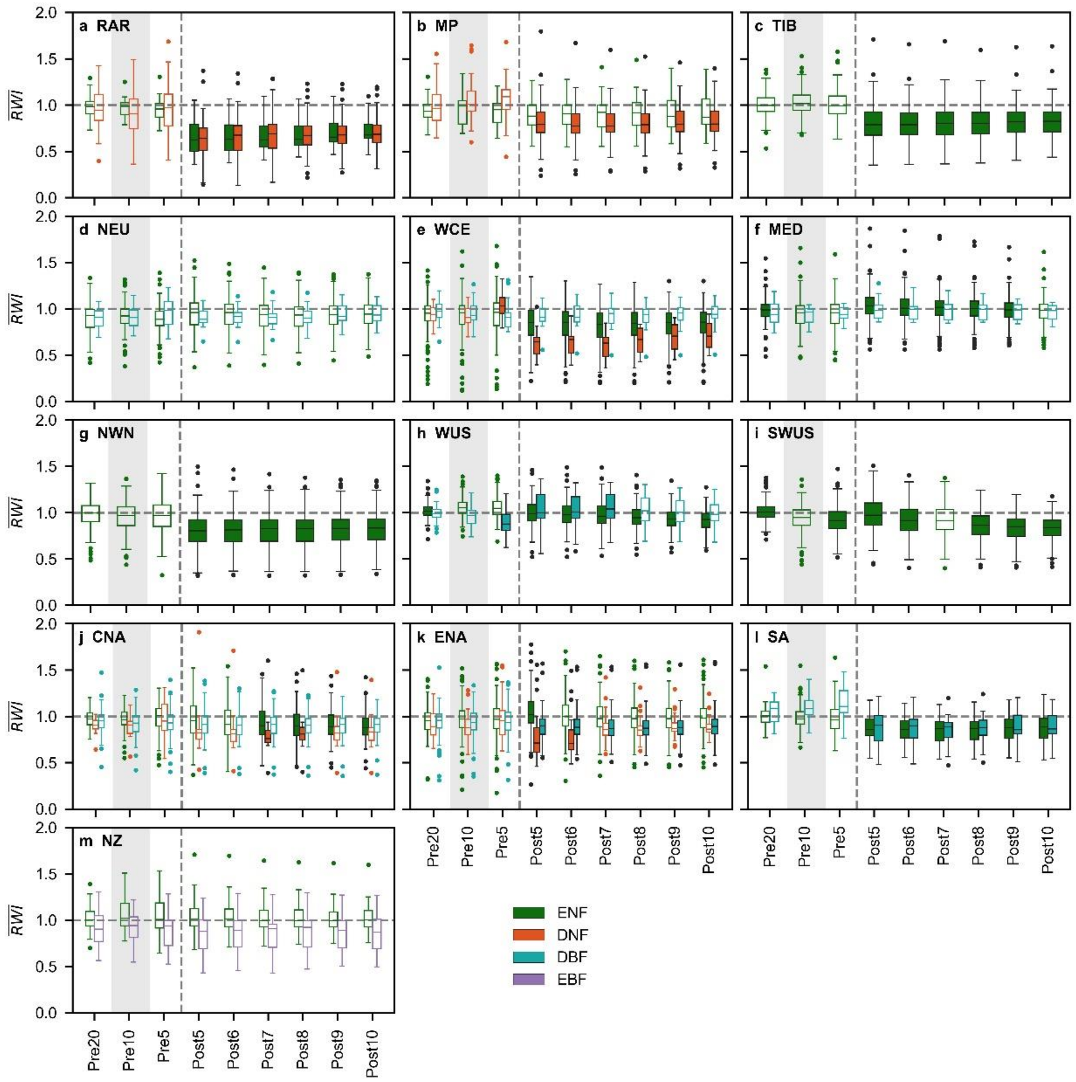

Supplementary Fig. 2 | Box plot of averaged ring-width index ($\overline{RWI}$) for different pre- and post-eruption periods in the 13 regions. Pre-eruption 20-, 10- and 5-years represent the period of 1789-1808, 1799-1808 and 1804-1808, respectively; post-eruption 5-, 6-, 7-, 8-, 9- and 10-years represent the period of 1815 to 1819, 1820, 1821, 1822, 1823 and 1824, respectively. In each panel, color filled boxes represent significant difference ($P < 0.05$) of tree growth relative to the pre-eruption 10-years (shaded in grey) estimated with the two-sided Wilcoxon test. On each box, the central bar indicates the median, the bottom and top edges indicate the 25th and 75th percentiles; the whiskers extend to all data points except outliers (which are plotted individually using dots). ENF, DNF, DBF and EBF represent evergreen needleleaf forests, deciduous needleleaf forests, deciduous broadleaf forests and evergreen broadleaf forests, respectively. RAR, NEU, WCE, MED, MP, TIB, NWN, WUS, SWUS, CNA, ENA, SA and NZ represent Russian-Arctic, northern Europe, western and central Europe, the Mediterranean region, the Mongolian Plateau, the Tibetan Plateau, northwestern North America, the west coast of US, the Southwestern U.S., central North America, eastern North America, the southern Andes and New Zealand, respectively. Exact p-values are provided in the source data: https://doi.org/10.11888/Terre.tpdc.300576.

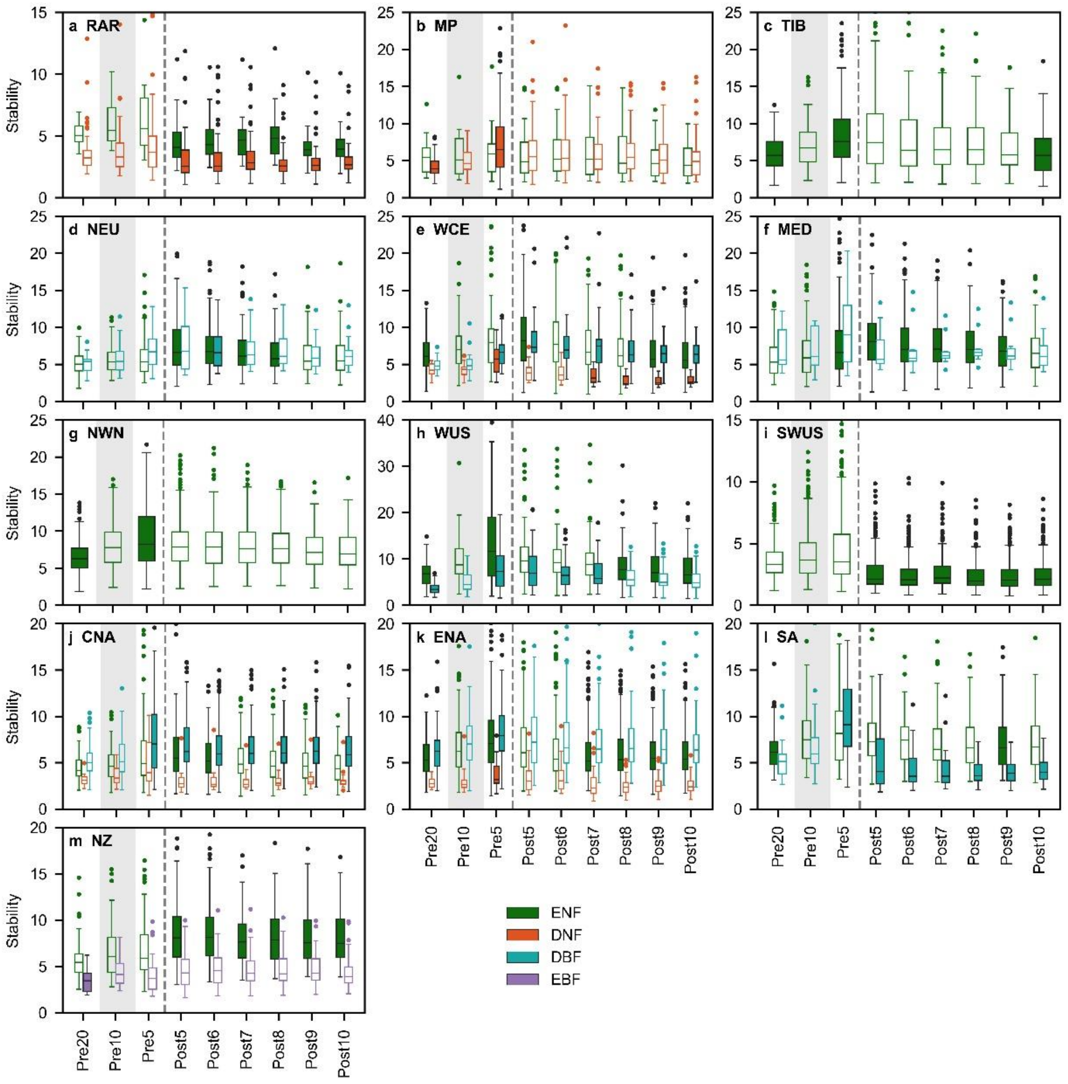

Supplementary Fig. 3 | Box plot of tree growth stability for different pre- and post-eruption periods in the 13 regions. Pre-eruption 20-, 10- and 5-years represent the period of 1789-1808, 1799-1808 and 1804-1808, respectively; post-eruption 5-, 6-, 7-, 8-, 9- and 10-years represent the period of 1815 to 1819, 1820, 1821, 1822, 1823 and 1824, respectively. In each panel, color filled boxes represent significant difference ($P < 0.05$) of tree growth stability relative to the pre-eruption 10-years (shaded in grey) estimated with the two-sided Wilcoxon test. On each box, the central bar indicates the median, the bottom and top edges indicate the 25th and 75th percentiles; the whiskers extend to all data points except outliers (which are plotted individually using dots). ENF, DNF, DBF and EBF represent evergreen needleleaf forests, deciduous needleleaf forests, deciduous broadleaf forests and evergreen broadleaf forests, respectively. RAR, NEU, WCE, MED, MP, TIB, NWN, WUS, SWUS, CNA, ENA, SA and NZ represent Russian-Arctic, northern Europe, western and central Europe, the Mediterranean region, the Mongolian Plateau, the Tibetan Plateau, northwestern North America, the west coast of US, the Southwestern U.S., central North America, eastern North America, the southern Andes and New Zealand, respectively. Exact p-values are provided in the source data: https://doi.org/10.11888/Terre.tpdc.300576.

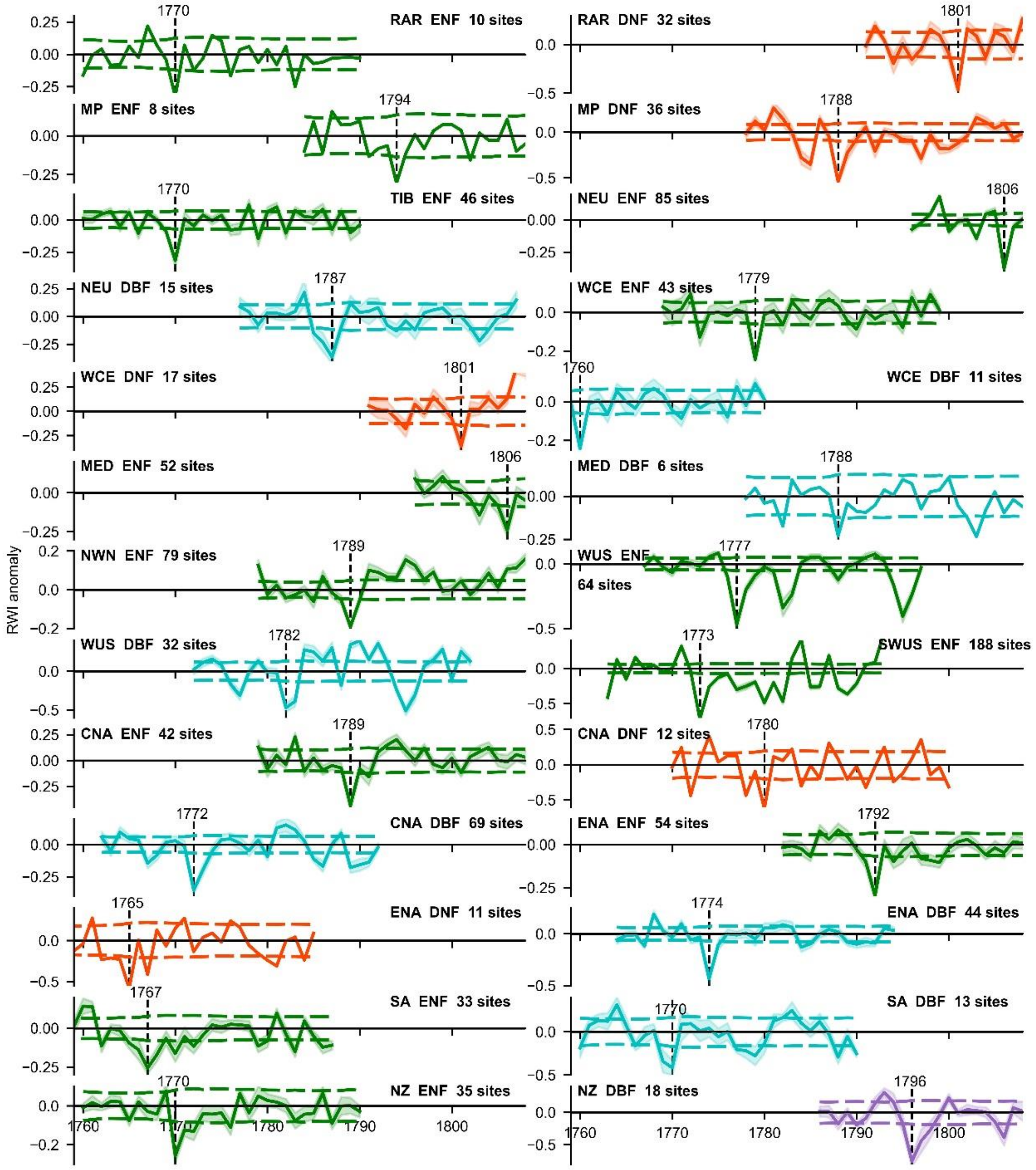

Supplementary Fig. 4 | Tree growth recovery baseline without volcanic forcing for each region in 1759 to 1808. The expected mean (solid lines) is bounded by the 95% confidence intervals (shaded horizontal boundary) for evergreen needleleaf forests (ENF, green lines), deciduous needleleaf forests (DNF, orange red lines) and deciduous broadleaf forests (DBF, cyan lines). The horizontal dashed lines indicate the threshold required for epochal anomalies to be statistically significant at the $\alpha = 0.05$ level. RAR, NEU, WCE, MED, MP, TIB, NWN, WUS, SWUS, CNA, ENA, SA and NZ represent Russian-Arctic, northern Europe, western and central Europe, the Mediterranean region, the Mongolian Plateau, the Tibetan Plateau, northwestern North America, the west coast of US, the Southwestern U.S., central North America, eastern North America, the southern Andes and New Zealand, respectively. Vertical dashed lines marked out the year when largest number of sites showed homogeneous growth decrease (i.e., RWI below -1σ).

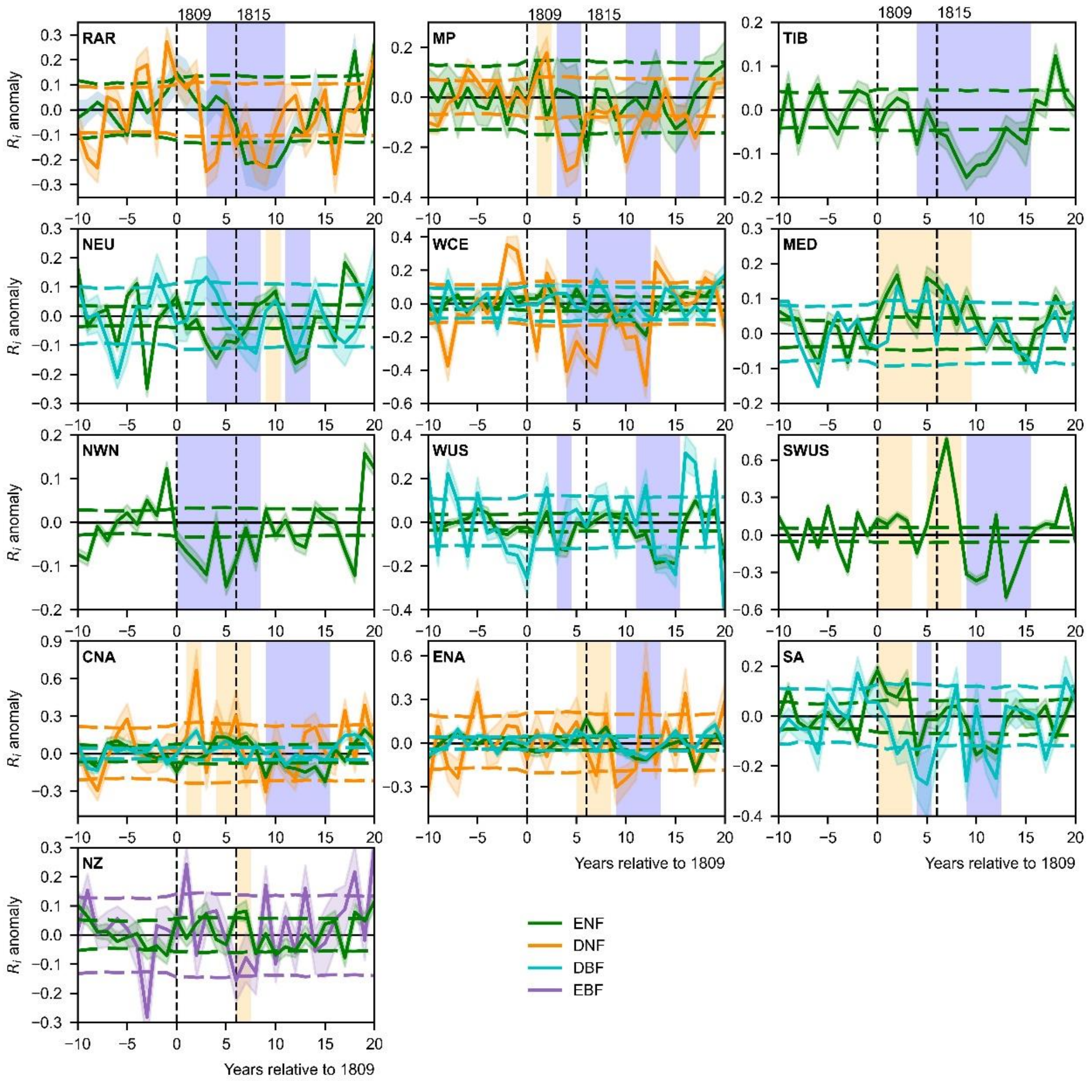

Supplementary Fig. 5 | SEA analysis of regional growth resilience (Ri) calculated using RWI detrended by a 30-years cubic smoothing spline after the 1809 and 1815 volcanic eruptions within selected regions. The expected mean (solid lines) is bounded by the 95% confidence intervals (shaded horizontal boundary) for evergreen needleleaf forests (ENF, green lines), deciduous needleleaf forests (DNF, orange red lines) and deciduous broadleaf forests (DBF, cyan lines). The horizontal dashed lines indicate the threshold required for epochal anomalies to be statistically significant at the $\alpha = 0.05$ level for different forest types. The purple and yellow shading mark the continuous periods when growth anomalies for either forest type were significant. RAR, NEU, WCE, MED, MP, TIB, NWN, WUS, SWUS, CNA, ENA, SA and NZ represent Russian-Arctic, northern Europe, western and central Europe, the Mediterranean region, the Mongolian Plateau, the Tibetan Plateau, northwestern North America, the west coast of US, the Southwestern U.S., central North America, eastern North America, the southern Andes and New Zealand, respectively. Vertical dashed lines marked out the eruption years.

Supplementary Table 1 | Number of tree-ring width chronologies within each study region

Forest types	Study regions												
	RAR	NEU	WCE	MED	MP	TIB	NWN	WUS	SWUS	CNA	ENA	SA	NZ
ENF	19	129	127	142	20	114	209	104	243	83	149	74	71
DNF	65	1	20	6	63	/	/	/	/	15	22	/	/
DBF	/	23	22	10	/	/	/	37	/	115	108	24	/
EBF	/	/	/	/	/	/	/	/	/	/	/	/	28

Note: Within each region, forest functional types with chronologies less than 10 were not included in the analysis. ENF, DNF, DBF and EBF represent evergreen needleleaf forests, deciduous needleleaf forests, deciduous broadleaf forests and evergreen broadleaf forests, respectively. RAR, NEU, WCE, MED, MP, TIB, NWN, WUS, SWUS, CNA, ENA, SA and NZ represent Russian-Arctic, northern Europe, western and central Europe, the Mediterranean region, the Mongolian Plateau, the Tibetan Plateau, northwestern North America, the west coast of US, the Southwestern U.S., central North America, eastern North America, the southern Andes and New Zealand, respectively.
