# Supplementary material for: Global tree growth resilience to cold extremes following the Tambora volcanic eruption
Source: Nat Commun. 2023 Oct 19;14:6616. doi: 10.1038/s41467-023-42409-w (PMC10587176; doi:10.1038/s41467-023-42409-w)
Supplement: Supplementary file 3 — Reporting Summary [file 41467_2023_42409_MOESM3_ESM.pdf]

nature portfolio

Corresponding author(s): Eryuan Liang

Last updated by author(s): Oct 7, 2023

Reporting Summary

Nature Portfolio wishes to improve the reproducibility of the work that we publish. This form provides structure for consistency and transparency in reporting. For further information on Nature Portfolio policies, see our Editorial Policies and the Editorial Policy Checklist.

Statistics

For all statistical analyses, confirm that the following items are present in the figure legend, table legend, main text, or Methods section.

n/a	Confirmed	
☐	☒	The exact sample size (*n*) for each experimental group/condition, given as a discrete number and unit of measurement
☒	☐	A statement on whether measurements were taken from distinct samples or whether the same sample was measured repeatedly
☐	☒	The statistical test(s) used AND whether they are one- or two-sided<br>*Only common tests should be described solely by name; describe more complex techniques in the Methods section.*
☐	☒	A description of all covariates tested
☐	☒	A description of any assumptions or corrections, such as tests of normality and adjustment for multiple comparisons
☐	☒	A full description of the statistical parameters including central tendency (e.g. means) or other basic estimates (e.g. regression coefficient) AND variation (e.g. standard deviation) or associated estimates of uncertainty (e.g. confidence intervals)
☐	☒	For null hypothesis testing, the test statistic (e.g. *F*, *t*, *r*) with confidence intervals, effect sizes, degrees of freedom and *P* value noted<br>*Give P values as exact values whenever suitable.*
☒	☐	For Bayesian analysis, information on the choice of priors and Markov chain Monte Carlo settings
☒	☐	For hierarchical and complex designs, identification of the appropriate level for tests and full reporting of outcomes
☒	☐	Estimates of effect sizes (e.g. Cohen's *d*, Pearson's *r*), indicating how they were calculated

Our web collection on statistics for biologists contains articles on many of the points above.

Software and code

Policy information about availability of computer code

Data collection	No software was used to collect data.
Data analysis	A modified double bootstrap Superposed Epoch Analysis (SEA) was used to evaluate the probability of the association between discrete volcanic eruptions and subsequent growth resilience shifts on regional scales. The algorithms for the modified double bootstrap SEA is obtained from "Rao, M. P. et al. A double bootstrap approach to Superposed Epoch Analysis to evaluate response uncertainty. Dendrochronologia. 55, 119-124 (2019)." Custom codes that are central to the research are written by R (version 4.2.2) and python (version 3.9). The custom codes for the analysis of the data are available from https://doi.org/10.11888/Terre.tpdc.300576.

For manuscripts utilizing custom algorithms or software that are central to the research but not yet described in published literature, software must be made available to editors and reviewers. We strongly encourage code deposition in a community repository (e.g. GitHub). See the Nature Portfolio guidelines for submitting code & software for further information.

Data

Policy information about availability of data

All manuscripts must include a data availability statement. This statement should provide the following information, where applicable:

- Accession codes, unique identifiers, or web links for publicly available datasets
- A description of any restrictions on data availability
- For clinical datasets or third party data, please ensure that the statement adheres to our policy

The International Tree-Ring Data Bank (ITRDB) is obtained from https://www.ncei.noaa.gov/pub/data/paleo/treering/measurements/, the GenTree

dendroecological collection is obtained from https://doi.org/10.6084/m9.figshare.c.4561037.v1, and the collection from the tree-ring group of the Institute of Tibetan Plateau Research, Chinese Academy of Sciences (ITPCAS) is available from https://doi.org/10.11888/Terre.tpdc.300576.

Research involving human participants, their data, or biological material

Policy information about studies with human participants or human data. See also policy information about sex, gender (identity/presentation), and sexual orientation and race, ethnicity and racism.

Reporting on sex and gender	Not relevant to this study.
Reporting on race, ethnicity, or other socially relevant groupings	Not relevant to this study.
Population characteristics	Not relevant to this study.
Recruitment	Not relevant to this study.
Ethics oversight	Not relevant to this study.

Note that full information on the approval of the study protocol must also be provided in the manuscript.

Field-specific reporting

Please select the one below that is the best fit for your research. If you are not sure, read the appropriate sections before making your selection.

☐ Life sciences ☐ Behavioural & social sciences ☒ Ecological, evolutionary & environmental sciences

For a reference copy of the document with all sections, see nature.com/documents/nr-reporting-summary-flat.pdf

Ecological, evolutionary & environmental sciences study design

All studies must disclose on these points even when the disclosure is negative.

Study description	In this study, we investigated the magnitude of forest resilience shifts and their recovery time to the strong and lasting volcanic cooling after the1809 and 1815 Tambora eruptions using 3304 tree-ring width chronologies across the globe. We calculated the changes in radial growth and stability for post-eruption 5 to 10 years and applied Superposed Epoch Analysis (SEA) to explore the timing and significance of growth departures from pre-eruption growth levels. We found that growth resilience were severely impacted by volcanic cooling in high latitudes and elevations especially in larch forests, and relatively mild influences for temperate forests. The recovery time extended longer in mid-latitudes than in high-latitudes, presumably due to they faced combined impacts of cold and drought stress.
Research sample	Tree-ring width chronologies were from existing datasets: the International Tree-Ring Data Bank (ITRDB), the GenTree dendroecological collection and the collection from the tree-ring group of the Institute of Tibetan Plateau Research, Chinese Academy of Sciences (ITPCAS). The sampling and processing procedure of tree-ring width data from the ITPCAS tree-ring group followed the basic principles of dendrochronology.
Sampling strategy	The sampling of tree-ring width data from the ITPCAS tree-ring group followed the principles of replication in dendrochronology. Each stand-level chronology was developed from multiple tree-level series (two cores from one tree, approximately 20 trees).
Data collection	The International Tree-Ring Data Bank (ITRDB) is obtained from https://www.ncei.noaa.gov/pub/data/paleo/treering/measurements/, the GenTree dendroecological collection is obtained from https://doi.org/10.6084/m9.figshare.c.4561037.v1, and the collection from the tree-ring group of the Institute of Tibetan Plateau Research, Chinese Academy of Sciences (ITPCAS) is obtained from https://doi.org/10.11888/Terre.tpdc.300576.
Timing and spatial scale	We used global tree-ring width chronologies spanning from 1759 to 1829.
Data exclusions	We retained global tree-ring width chronologies that date back to at least 1759.
Reproducibility	All attempts to repeat the analysis with these data and statistical methods would be successful.
Randomization	Not relevant to this study. Tree-ring data are observational data under natural conditions not from manipulative experiments.
Blinding	Not relevant to this study. Investigators who have contributed tree-ring data followed the basic principles of site selection and replication.

Did the study involve field work? ☐ Yes ☒ No

Reporting for specific materials, systems and methods

We require information from authors about some types of materials, experimental systems and methods used in many studies. Here, indicate whether each material, system or method listed is relevant to your study. If you are not sure if a list item applies to your research, read the appropriate section before selecting a response.

Materials & experimental systems

n/a	Involved in the study	
☒	☐	Antibodies
☒	☐	Eukaryotic cell lines
☒	☐	Palaeontology and archaeology
☒	☐	Animals and other organisms
☒	☐	Clinical data
☒	☐	Dual use research of concern
☒	☐	Plants

Methods

n/a	Involved in the study	
☒	☐	ChIP-seq
☒	☐	Flow cytometry
☒	☐	MRI-based neuroimaging
